# Effects of mandibular advancement devices vs. CPAP on blood pressure in obstructive sleep apnea: a systematic review and meta-analysis of randomized controlled trials

**DOI:** 10.3389/fneur.2026.1846726

**Published:** 2026-05-20

**Authors:** Ting Cheng, Qiang Wang, Wei Wei

**Affiliations:** 1 Department of Pharmacy, Deyang Stomatological Hospital, Deyang, Sichuan, China; 2 Department of Orthodontics, Deyang Stomatological Hospital, Deyang, Sichuan, China; 3 Department of Pharmacy, People's Hospital of Zhongjiang County, Deyang, Sichuan, China

**Keywords:** continuous positive airway pressure, hypertension, mandibular advancement device, meta-analysis, obstructive sleep apnea syndrome, systematic review

## Abstract

**Background:**

Continuous positive airway pressure (CPAP) is the gold standard for obstructive sleep apnea (OSA), but its clinical effectiveness in cardiovascular risk management is often limited by suboptimal adherence.

**Objective:**

To systematically evaluate and compare the effects of mandibular advancement devices (MAD) vs. CPAP and inactive controls on blood pressure (BP), sleep-related respiratory events, and treatment adherence in patients with OSA.

**Methods:**

We systematically searched PubMed, Embase, Cochrane Library, and Web of Science from database inception to February 2026. RCTs reporting ambulatory BP outcomes pre- and post-MAD treatment were included. Secondary outcomes included the apnea-hypopnea index (AHI), Epworth Sleepiness Scale (ESS), and objective treatment adherence. Data were pooled using mean differences (*MD*) and 95% confidence intervals (CIs).

**Results:**

Fourteen independent RCTs (comprising 16 reports) encompassing a total of 1,141 patients, met the inclusion criteria. Compared with inactive controls, MAD showed overall trends of BP reduction. In head-to-head comparisons with CPAP, MAD demonstrated comparable cardiovascular benefits on 24-h and nighttime BP parameters. Notably, MAD achieved a significantly greater reduction in daytime systolic BP compared with CPAP (*MD* = −1.99 mmHg, 95% CI: −3.82 to −0.17; *p* = 0.03). While CPAP demonstrated superior physiological efficacy in reducing AHI (*MD* = 8.45 events/h, *p* < 0.001), MAD and CPAP yielded comparable improvements in subjective sleepiness (ESS). Crucially, pooled objective tracking data revealed that MAD had significantly longer nightly adherence than CPAP (*MD* = 0.71 h/night, 95% CI: 0.30 to 1.13; *p* < 0.001).

**Conclusion:**

Despite a physiological inferiority in reducing AHI, MAD appears to offer cardiovascular benefits comparable to CPAP and demonstrates a statistically significant reduction in daytime SBP, which may be partially facilitated by its superior objective adherence. Therefore, for OSA patients who cannot tolerate CPAP, MAD may serve as a viable alternative option for cardiovascular risk management.

**Systematic review registration:**

https://www.crd.york.ac.uk/PROSPERO/view/CRD420261303916, identifier: CRD420261303916.

## 1 Introduction

Hypertension is a major risk factor for cardiovascular and cerebrovascular diseases (1). Although pharmacotherapy remains the cornerstone of blood pressure management, obstructive sleep apnea (OSA) is increasingly recognized as an important, underdiagnosed, and modifiable cause of hypertension (2). In patients with OSA, repeated upper airway collapse during sleep leads to intermittent hypoxia, increased sympathetic nervous system activity, and transient elevations in blood pressure (3). Consequently, current hypertension prevention and treatment guidelines (4, 5) and scientific statements (6) all recommend screening hypertensive patients for OSA and providing appropriate treatment upon diagnosis.

Continuous positive airway pressure (CPAP) is recommended as the first-line treatment for OSA, typically delivering automatically adjusted positive pressure through a nasal cannula or nasal mask to maintain upper airway patency during sleep. However, many patients refuse treatment or have difficulty adhering to it, particularly those without symptoms of excessive daytime sleepiness (7, 8).

As a primary alternative to CPAP, mandibular advancement devices (MADs)—oral appliances that advance the lower jaw—have in recent years been recommended for the treatment of mild-to-moderate OSA, as well as for severe cases where patients are intolerant of CPAP (9–11).

Although MADs are generally slightly less effective than CPAP in reducing the apnea-hypopnea index (AHI), a growing body of research suggests that MADs may be non-inferior to CPAP in improving health outcomes, including blood pressure control (12, 30). However, the specific effects of MADs on blood pressure remain controversial and uncertain. Some recent systematic reviews have noted that the effect of MAD on diastolic blood pressure may not be statistically significant or that its blood pressure-lowering effect is significantly influenced by treatment duration, circadian rhythms, and device design (e.g., custom-made vs. non-custom-made). While previous meta-analyses have explored the effects of MAD and CPAP on blood pressure (13), they were largely constrained by a pre-dominance of mild-to-moderate OSA, a high proportion of normotensive patients (which dilutes the blood pressure-lowering effect due to a “floor effect”) (14), and a reliance on subjective self-reported adherence data (15). Recently, paradigm-shifting randomized controlled trials—such as the CRESCENT trial and the CHOICE trial—have been published (16, 30). These contemporary trials expanded the evidence base across the OSA severity spectrum—including severe OSA patients with high cardiovascular risk—while utilizing embedded microsensors to provide precise, objective adherence data.

In light of this, the present study aims to comprehensively evaluate the effects of MAD on blood pressure in OSA patients through a systematic review and meta-analysis, thereby providing more robust evidence-based medical evidence to inform clinical decision-making.

## 2 Materials and methods

### 2.1 Retrieval strategy

This systematic review and meta-analysis strictly adhered to the “Preferred Reporting Items for Systematic Reviews and Meta-Analyses” (PRISMA) guidelines (17). We searched the Cochrane Library, PubMed, Embase, and Web of Science databases, with a search period ranging from the inception of each database to February 2026. English search terms included: “obstructive sleep apnea”, “mandibular advancement device”, “blood pressure”, and “randomized controlled trial”. The specific search strategy is provided in Supplementary material. The study protocol has been registered on the PROSPERO International Registry of Systematic Reviews (PROSPERO: CRD420261303916). In addition, the reference lists of all included studies and relevant systematic reviews were manually screened to identify any further eligible trials.

### 2.2 Literature inclusion and exclusion criteria

Develop screening criteria based on the PICO principles (Population, Intervention, Comparison, Outcome, and Study design) (18).

1) Participants: Adult patients (aged ≥18 years) diagnosed with OSA via polysomnography (PSG) or home sleep monitoring.

2) Interventions: Use of various types of custom or non-custom mandibular advancement devices.

3) Comparisons: Includes CPAP, sham orthotics (Sham OA/inactive orthotics), and conservative treatments (e.g., lifestyle interventions).

4) Outcomes: Pre- and post-treatment blood pressure data [systolic blood pressure (SBP), diastolic blood pressure (DBP), or mean arterial pressure (MAP)] must be reported. Secondary outcomes include the AHI, Epworth Sleepiness Scale (ESS) scores, and objective treatment adherence (h/night).

5) Study design: randomized controlled trials (RCTs).

Exclusion criteria: review articles, case reports, animal studies, studies involving only patients with simple snoring, and studies from which standard deviation (SD) data cannot be extracted.

### 2.3 Literature screening and data extraction

Two researchers (TC and QW) independently conducted literature searches, data extraction, and screening, cross-checked each other's work, and submitted studies with discrepancies to a third researcher (WW) for analysis to determine eligibility. We collected key characteristics from each study meeting the inclusion criteria. These included: (1) Study characteristics: first author, year of publication, country, and study design type; (2) Demographic characteristics of participants: sample size, age, sex, BMI, baseline AHI, and baseline blood pressure status; (3) Intervention details: type of MAD (e.g., custom-made vs. non-custom-made) and control measures (CPAP, placebo, or no treatment); (4) Outcome measures: Primary outcomes included mean changes in systolic blood pressure, diastolic blood pressure, and mean arterial pressure, stratified by measurement period (24-h, daytime, and nighttime). Secondary outcomes included AHI, changes in ESS scores, and treatment adherence.

### 2.4 . Quality assessment

Two reviewers (TC and QW) conducted the assessment using the Cochrane Risk of Bias in Interventions (ROB-II) tool (19). Any disagreements during the assessment were resolved through discussion or consultation with a third reviewer (WW). The RoB 2 tool categorizes the quality of randomized controlled trials into five domains (randomization process, bias in allocation to the intended intervention, missing outcome data, measurement of outcomes, and selection of outcomes to report), thereby providing a structured overall assessment. All eligible randomized controlled trials were included in our meta-analysis regardless of study quality.

### 2.5 Data synthesis and heterogeneity assessment

The analysis made use of pooled statistics, namely the mean difference (*MD*). For crossover trials, end-of-treatment data from each period were treated as parallel-group data, assuming minimal carryover effects following adequate washout periods. For all continuous outcomes, post-treatment values were consistently used for pooling rather than change-from-baseline scores. For continuous outcomes, a negative *MD* indicates a greater reduction in the parameter (e.g., lower blood pressure or ESS score) favoring the MAD group, whereas a positive *MD* indicates a greater reduction favoring the CPAP group. Statistical heterogeneity among the included studies was evaluated using the *I*^2^ statistic. An *I*^2^ value of ≤ 50% indicates acceptable heterogeneity, prompting the use of a fixed-effect model for data pooling. Conversely, an *I*^2^ value > 50% indicates substantial heterogeneity, in which case a random-effects model was employed. Egger's test was used to evaluate publication bias, accompanied by a funnel plot for visual inspection. Sensitivity analyses were carried out to evaluate the reliability of the results. A *p*-value less than 0.05 was deemed statistically significant. Stata (version 16.1, StataCorp) and Review Manager (RevMan, version 5.4.1) were used for all data analyses.

## 3 Results

### 3.1 Results of the literature search and study selection

**Figure 1:**
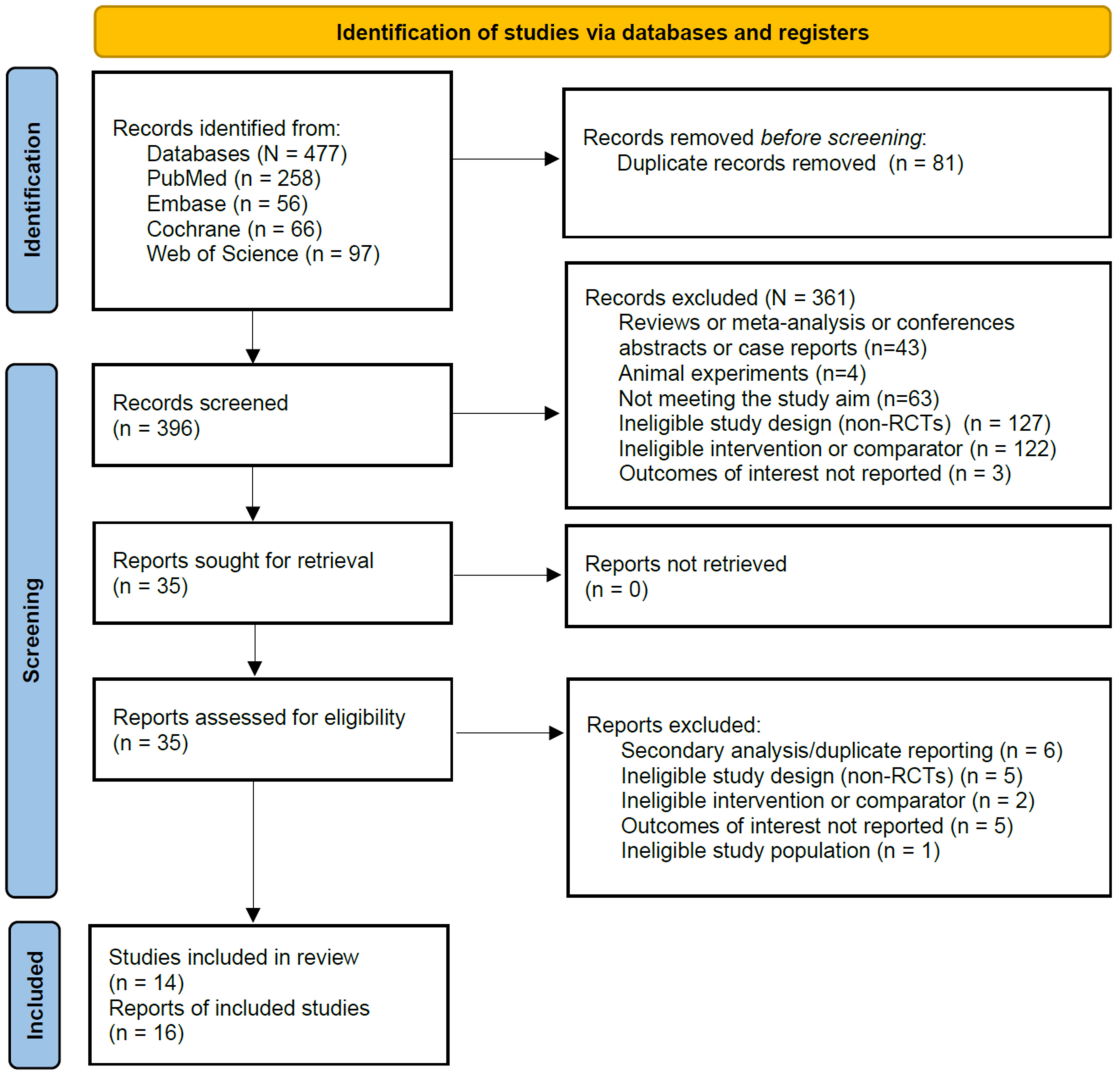
PRISMA flow diagram of the literature search and study selection process. PRISMA, preferred reporting items for systematic reviews and meta-analyses.

We conducted a comprehensive search of all databases, identifying a total of 477 records. After removing duplicates, 396 records remained. Following screening of titles and abstracts, 361 records were excluded, leaving 35 studies for full-text evaluation. Of these, 18 studies were excluded for the following reasons: incorrect study design (intervention, control, or population; *n* = 3); failure to report primary outcome measures (*n* = 5); exclusion due to non-compliance with study design criteria (non-RCT; *n* = 5); and secondary analyses or duplicate reporting of data (*n* = 6). Ultimately, 14 independent studies (corresponding to 16 research reports/publications) met all inclusion criteria and were included in this systematic review and meta-analysis (Figure 1).

### 3.2 Study characteristics

This meta-analysis included 14 independent randomized controlled trials (RCTs, corresponding to 16 publications) (16, 20–34), encompassing a total of 1,141 patients. The included studies comprising 7 crossover designs and 7 parallel-group designs, with follow-up periods ranging from 4 weeks to 12 months (see Table 1 for details). Participants primarily consisted of individuals with OSA ranging from mild to severe at baseline. Notably, several key studies specifically enrolled subgroups with confirmed hypertension or high cardiovascular risk (e.g., Andrén et al. and the recent CRESCENT trial), providing high-quality evidence for evaluating targeted antihypertensive effects. Regarding intervention and control settings, all experimental groups used MADs, while control groups included inactive controls (dummy devices/placebos/conservative treatment) and standard active controls (CPAP).

**Table 1:** Baseline characteristics of the included randomized controlled trials. (30).

Study references	Country	Study design	*N* (randomized/ analyzed)	Male, %	Baseline characteristics (age, BMI, AHI)	Interventions (experimental vs. control)	Follow-up DURATION	BP measurement method	Outcomes
(21)	Australia	Crossover RCT	104/80	78.8%	Age: 46.4 BMI: 31.0 AHI: 21.5	ARBMAD vs. CPAP vs. Placebo	Crossover, 3 months each	24 h ABPM	BP: 24 h, asleep SBP/DBP Sleep/symptoms: AHI, ESS
(26)	Australia	Crossover RCT	75/67	79.1%	Age: 48.0 BMI: 29.2 AHI: 27.0	MAD vs. Sham OA	Crossover, 4 weeks each	24 h ABPM	BP: 24 h, awake, asleep SBP/DBP Sleep/symptoms: AHI, ESS
(28)	China	Parallel RCT	101/91	Not Reported	Age: 46.0 BMI: 27.4 AHI: 21.4	MAD vs. CPAP vs. Control	10 weeks	Office BP	BP: office SBP/DBP Sleep/symptoms: AHI, ESS
(20)	Sweden	Parallel RCT	72/72	79.2%	Age: 58.0 BMI: 30.0 AHI: 20.0	MAD vs. Sham OA	3 months	24 h ABPM	BP: 24 h, awake, asleep SBP/DBP/MAP Sleep/symptoms: AHI, ESS
(23, 31)	Australia	Crossover RCT	126/108	81.0%	Age: 49.5 BMI: 29.5 AHI: 25.6	MAD vs. CPAP	Crossover, 1 month each	24 h ABPM	BP: 24 h, awake, asleep SBP/DBP Sleep/symptoms: AHI, ESS
(22)	Brazil	Crossover RCT	39/29	82.8%	Age: 47.0 BMI: 28.4 AHI: 42.3	MAD vs. CPAP vs. Sham	Crossover, 1 month each	24 h ABPM	BP: 24 h, awake, asleep SBP/DBP Sleep/symptoms: AHI, ESS
(29, 32)	Sweden	Parallel RCT	96/85	67.7%	Age: 52.0 BMI: 27.6 AHI: 17.0	MAD vs. Sham OA	4 months	24 h ABPM	BP: daytime, nighttime SBP/DBP Sleep/symptoms: AHI, ESS
(25)	Germany	Crossover RCT	48/40	Not Reported	Age: 54.0 BMI: 30.0 AHI: 25.0	MAD vs. CPAP	Crossover, 12 wks each	Office BP	BP: Office SBP/DBP Sleep/symptoms: AHI, ESS
(24)	France	Parallel RCT	150/150	85.6%	Age: 53.8 BMI: 27.0 AHI: 41.0	MAD vs. Sham OA	2 months	Office BP	BP: Office SBP/DBP Sleep/symptoms: AHI, ESS
(43)	Japan	Crossover RCT	45/40	75.0%	Age: 54.9 BMI: 26.3 AHI: 28.6	MAD vs. CPAP	Crossover, 4 wks each	24 h ABPM	BP: 24 h, awake, sleep SBP/DBP/MAP sleep/symptoms: REI, ESS
(27)	Brazil	Parallel RCT	79/58	54.4%	Age: 46.0 BMI: 28.0 AHI: 9.3	MAD vs. CPAP vs. Control	12 months	24 h ABPM	BP: 24 h, awake, asleep SBP/DBP Sleep/symptoms: AHI, ESS
(33)	Netherlands	Parallel RCT	85/54	82.4%	Age: 50.7 BMI: 30.2 AHI: 20.9	MAD vs. CPAP	12 months	24 h ABPM	BP: 24 h, daytime, nighttime SBP/DBP Sleep/symptoms: AHI, ESS
(32) (CRESCENT)	Singapore	Parallel RCT (non-inferiority)	220/199	85.5%	Age: 61.0 BMI: 27.5 AHI: 38.0	MAD vs. CPAP	6 months	24 h ABPM	BP: 24 h, awake, asleep SBP/DBP/MAP Sleep/symptoms: AHI, ESS
(16) (CHOICE)	Canada	Crossover RCT	79/68	73.4%	Age: 52.3 BMI: 28.5 REI: 21.1	MAD vs. CPAP	Crossover, 1 month each	Office BP	BP: office SBP/DBP Sleep/symptoms: REI, ESS

SBP, systolic blood pressure; DBP, diastolic blood pressure; MAP, mean arterial pressure; ABPM, ambulatory blood pressure monitoring; AHI, Apnea-Hypopnea Index; REI, Respiratory Event Index; ESS, Epworth Sleepiness Scale; MAD, mandibular advancement device; CPAP, continuous positive airway pressure; OA, oral appliance.

### 3.3 Literature quality assessment

**Figure 2:**
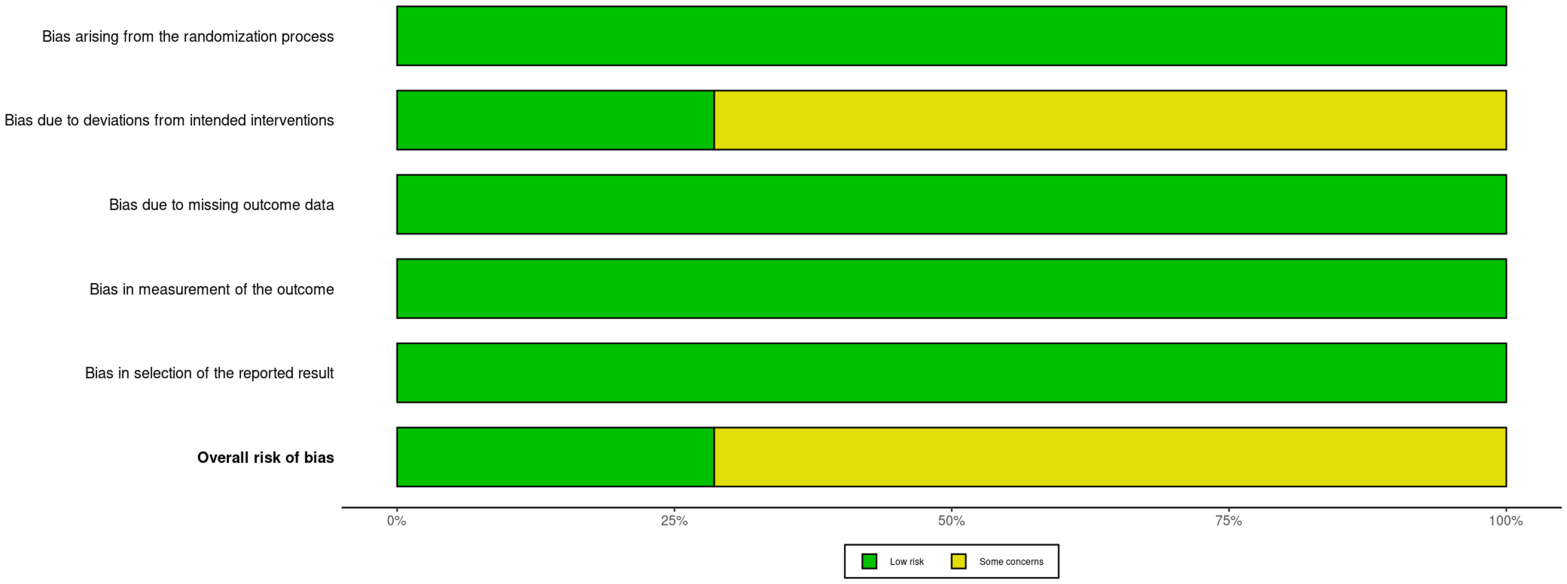
Risk of bias graph.

**Figure 3:**
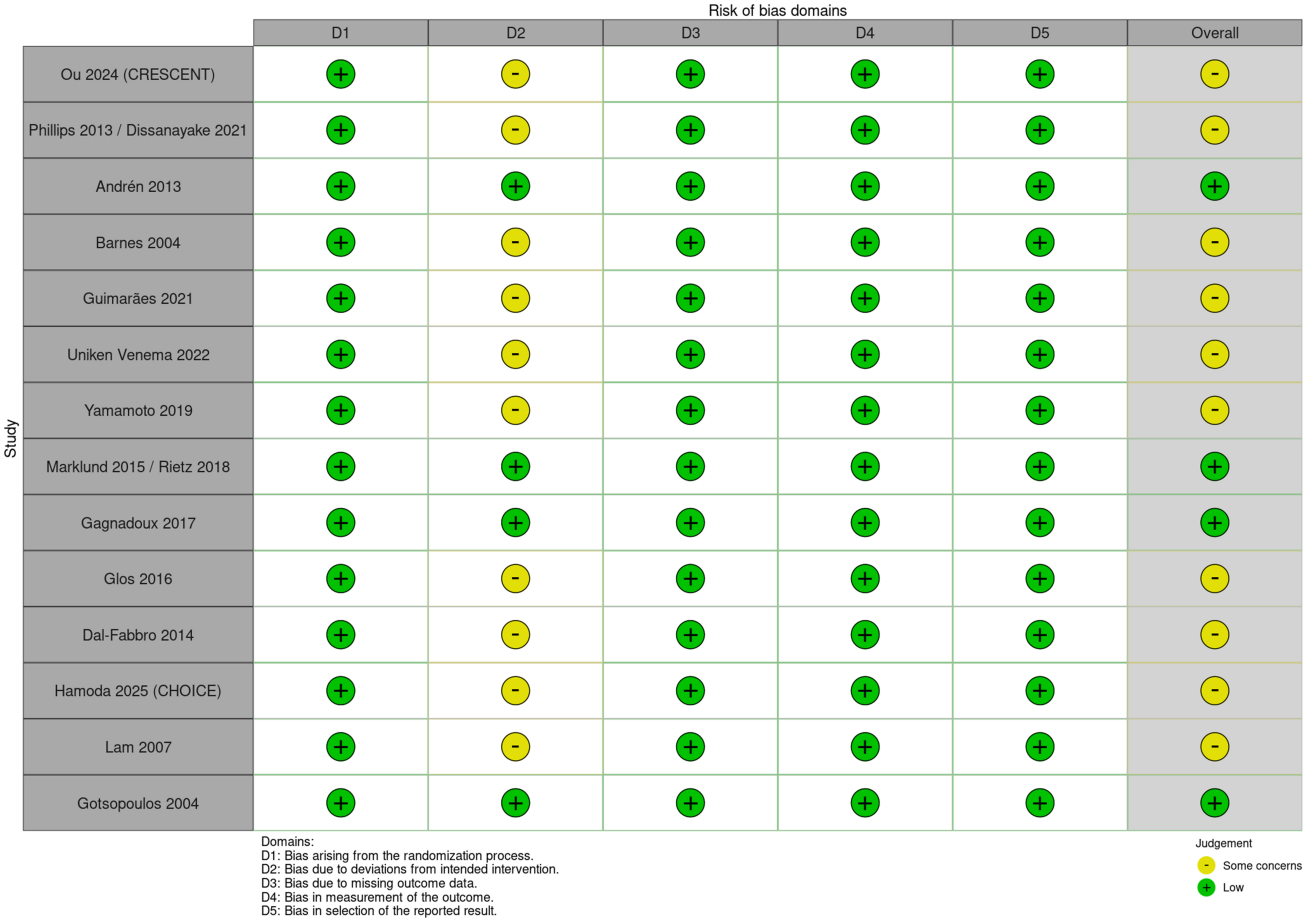
Risk of bias summary: review authors' judgements about each risk of bias item for each included study.

This meta-analysis conducted a rigorous methodological evaluation of the included studies. The overall percentage of risk of bias items across all studies is summarized in Figure 2 (Risk of bias graph), and the specific risk of bias assessment results for each individual study are detailed in Figure 3 (Risk of bias summary).

### 3.4 Meta-analysis results

#### 3.4.1 Effects on blood pressure outcomes

In comparisons between MAD and inactive controls, pooled analyses revealed overall trends toward reduction across all evaluated blood pressure parameters—including 24-h, daytime, and nighttime systolic and diastolic blood pressures—although these reductions did not reach statistical significance (all *p* > 0.05; Supplementary Figures S1–S6).

**Figure 4:**
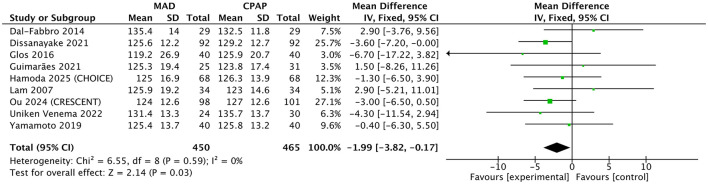
Forest plot of daytime systolic blood pressure MAD therapy vs. CPAP. Data are presented as pooled mean differences (*MD*) with 95% confidence intervals (CIs) using an inverse-variance fixed-effects model. MAD, mandibular advancement device; CPAP, continuous positive airway pressure; SD, standard deviation; IV, inverse variance; CI, confidence interval.

When compared with CPAP, MAD demonstrated comparable cardiovascular benefits for most ambulatory blood pressure parameters. There were no statistically significant differences between MAD and CPAP regarding 24-h SBP/DBP, nighttime SBP/DBP, and daytime DBP (all *p* > 0.05; Supplementary Figures S7–S11). Notably, however, a pooled analysis of nine studies demonstrated that MAD achieved a statistically significant greater reduction in daytime SBP compared with CPAP (*MD* = −1.99 mmHg, 95% CI: −3.82 to −0.17; *p* = 0.03; *I*^2^ = 0%; Figure 4).

#### 3.4.2 Effects on sleep-related respiratory events and daytime sleepiness

**Figure 5:**
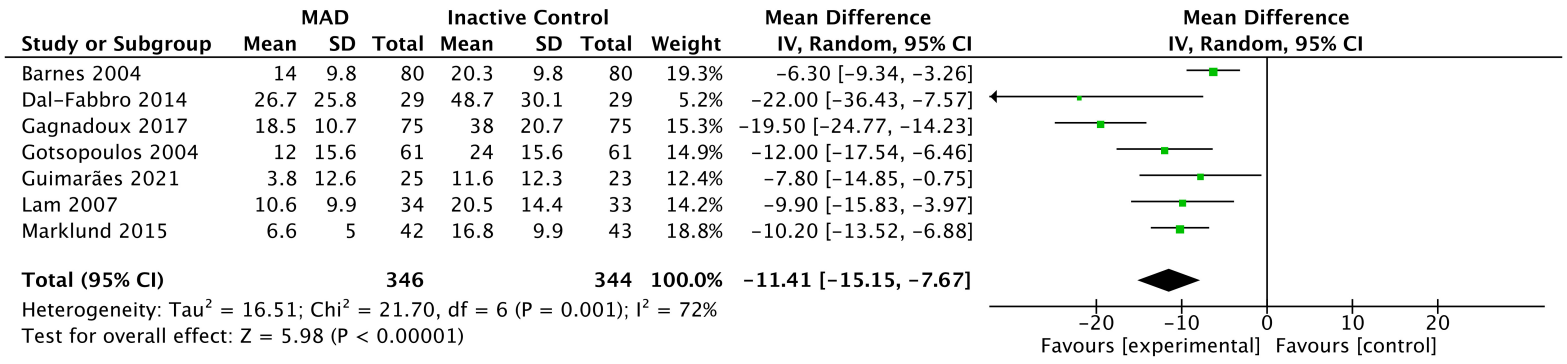
Forest plot of AHI comparing MAD therapy vs. inactive control. Data are presented as pooled mean differences (*MD*) with 95% confidence intervals (CIs) using an inverse-variance random-effects model. MAD, mandibular advancement device; AHI, apnea-hypopnea index; SD, standard deviation; IV, inverse variance; CI, confidence interval.

**Figure 6:**
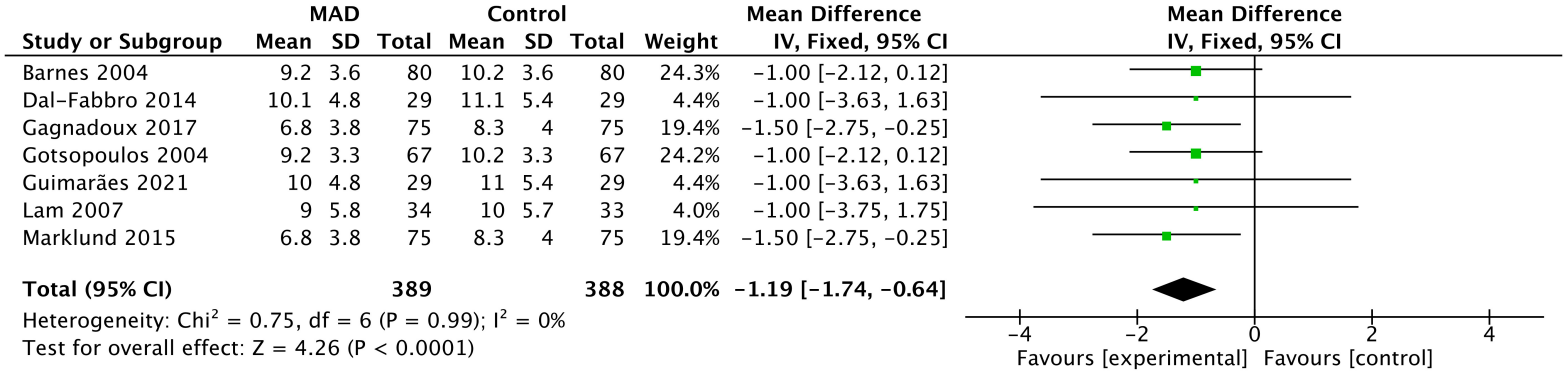
Forest plot of ESS comparing MAD therapy vs. inactive control. Data are presented as pooled mean differences (*MD*) with 95% confidence intervals (CIs) using an inverse-variance fixed-effects model. MAD, mandibular advancement device; ESS, Epworth Sleepiness Scale; SD, standard deviation; IV, inverse variance; CI, confidence interval.

**Figure 7:**
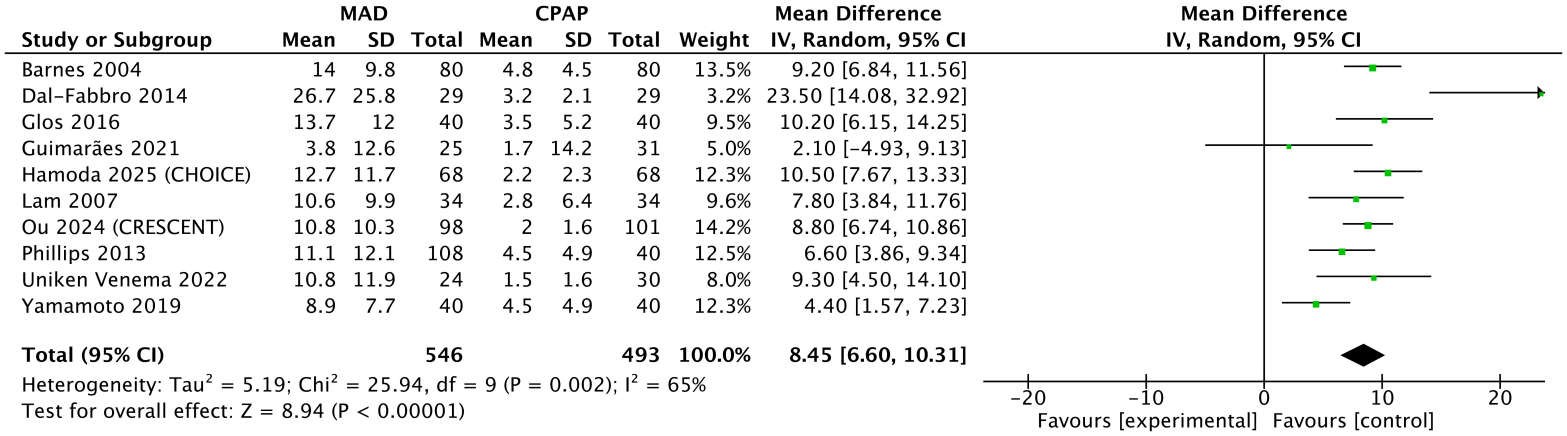
Forest plot of AHI comparing MAD therapy vs. CPAP. Data are presented as pooled mean differences (*MD*) with 95% confidence intervals (CIs) using an inverse-variance random-effects model. MAD, mandibular advancement device; CPAP, continuous positive airway pressure; AHI, apnea-hypopnea index; SD, standard deviation; IV, inverse variance; CI, confidence interval.

Compared with inactive controls, MAD significantly reduced the AHI (7 studies; *MD* = −11.41 events/h, 95% CI: −15.15 to −7.67; *p* < 0.001; Figure 5) and significantly improved daytime sleepiness as measured by the ESS (7 studies; *MD* = −1.19, 95% CI: −1.74 to −0.64; *p* < 0.001; Figure 6). However, in direct head-to-head comparisons with CPAP, CPAP demonstrated significantly greater physiological efficacy in reducing AHI (10 studies; *MD* = 8.45 events/h, 95% CI: 6.60 to 10.31; *p* < 0.001; *I*^2^ = 65%; Figure 7). Despite this physiological inferiority, MAD showed a comparable effect to CPAP in improving subjective daytime sleepiness, with no significant difference observed in ESS reduction between the two therapies (8 studies; *MD* = −0.03, 95% CI: −0.55 to 0.50; *p* = 0.92; *I*^2^ = 0%; Supplementary Figure S12).

#### 3.4.3 Objective treatment adherence

**Figure 8:**
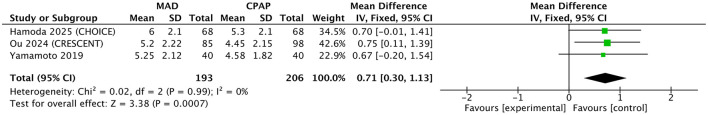
Forest plot of objective treatment adherence (h/night) comparing MAD therapy vs. CPAP. Data are presented as pooled mean differences (*MD*) with 95% confidence intervals (CIs) using an inverse-variance fixed-effects model. MAD, mandibular advancement device; CPAP, continuous positive airway pressure; SD, standard deviation; IV, inverse variance; CI, confidence interval.

Three studies provided objective adherence data using tracking monitors. The pooled results highlighted a significant compliance advantage for MAD, showing that MAD was associated with significantly longer objective nightly usage compared with CPAP (*MD* = 0.71 h/night, 95% CI: 0.30 to 1.13; *p* < 0.001; *I*^2^ = 0%; Figure 8).

### 3.5 Publication bias

**Figure 9:**
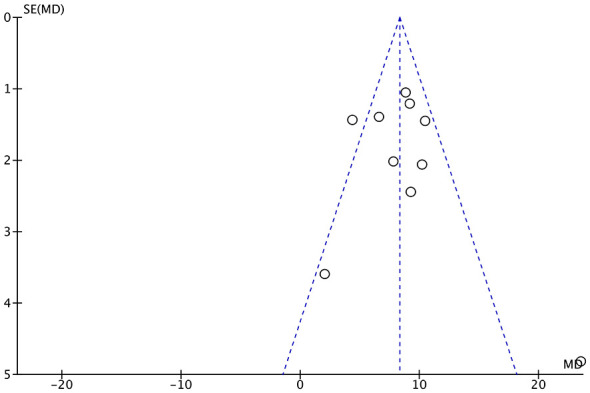
Funnel plot of the AHI in the comparison of MAD vs. CPAP. Data points represent individual studies plotted by their mean difference (*MD*) against the standard error (SE) of the *MD*. MAD, mandibular advancement device; CPAP, continuous positive airway pressure; AHI, apnea-hypopnea index; *MD*, mean difference; SE, standard error.

According to the Cochrane Handbook guidelines, an assessment of publication bias is recommended only when at least 10 studies are available for a given comparison. Consequently, publication bias was evaluated specifically for the AHI outcome in the MAD vs. CPAP comparison (*n* = 10). Visual inspection of the funnel plot revealed a symmetrical, inverted funnel shape, suggesting no substantial asymmetry (Figure 9). Furthermore, the objective Egger's test confirmed the absence of significant publication bias (*p* = 0.184).

### 3.6 Sensitivity analysis

A leave-one-out sensitivity analysis was conducted for the primary significant outcomes, including the reduction in daytime SBP and objective treatment adherence. By sequentially excluding one study at a time and recalculating the pooled effect sizes, we found that the omission of any single study did not substantially alter the overall *MD* or the statistical significance. This indicates that our core findings are highly robust and not driven by any individual trial.

## 4 Discussion

This meta-analysis included 14 randomized controlled trials (16 reports in total) comparing the effects of MAD therapy on blood pressure in patients with OSA. The results showed that, compared with the no-intervention control group, the MAD group exhibited a downward trend in 24-h systolic blood pressure SBP and DBP; compared with CPAP, MAD even demonstrated a specific advantage in reducing daytime SBP. Furthermore, MAD significantly reduced the AHI in OSA patients and significantly improved daytime sleepiness symptoms ESS. In terms of treatment adherence, the objective average nightly usage time for MAD was 0.71 h longer than that for CPAP. Overall, this study indicates that MAD effectively improves clinical symptoms and provides tangible cardiovascular benefits (such as blood pressure control). These cardiovascular benefits might be partially explained by the observed trend of higher objective treatment adherence with MAD. Although objective adherence data were only available in three recent trials, this finding supports the “efficacy-effectiveness balance” hypothesis.

Mechanistically, the blood pressure-lowering effects of MAD are are closely linked to the elimination of intermittent hypoxia (IH). Recent studies demonstrate that IH induces hypertension via p300/CBP-mediated HIF-1α acetylation and subsequent Nox-dependent oxidative stress (35). Furthermore, IH triggers endothelial dysfunction and systemic inflammation in peripheral blood mononuclear cells through the NF-κB and HIF-1 pathways (36). By physically expanding and stabilizing the upper airway, MAD effectively relieves IH, thereby directly downregulating these pathogenic molecular cascades. This targeted attenuation of sympathetic overactivation and inflammation provides a precise rationale for the cardiovascular benefits observed in our meta-analysis.

The findings of this study are generally consistent with the results of previous studies and provide updated evidence-based medical evidence. Early network meta-analyses and the study by Sharples et al. suggest that although CPAP is superior to MAD in terms of absolute physiological efficacy in reducing AHI, there is no significant difference between the two in terms of overall blood pressure-lowering effects and improvement of subjective symptoms (13, 37). In recent years, a meta-analysis by Belanche-Monterde et al. (38) has further supported the finding that MAD can induce a mild decrease in SBP and that the extent of blood pressure reduction (particularly nocturnal SBP) is positively correlated with treatment duration. This study not only confirms the consistency between MAD and CPAP in terms of the trend toward nocturnal SBP reduction but also highlights, for the first time, the advantage of MAD in reducing daytime SBP.

This coexistence of “physiological efficacy disadvantage” and “clinical benefit equivalence (or even superiority)” perfectly aligns with the “efficacy-effectiveness balance” theory (39): CPAP's greater physiological advantages are offset by its lower wear rate, whereas MAD's higher compliance (this study confirmed an additional 0.71 h of use per night) compensates for its absolute efficacy deficit, ultimately translating into cardiovascular benefits comparable to or even superior to those of CPAP.

However, this study reached conclusions that do not fully align with some previous studies on certain key endpoints, highlighting its clinical value. Some previous studies have suggested that the effect of MAD on 24-h ambulatory blood pressure is extremely limited or that no statistically significant differences were observed (24). These previous studies often suffered from a “floor effect” due to the inclusion of a large number of patients with mild to moderate sleep apnea and normal baseline blood pressure, thereby underestimating the blood pressure-lowering potential of MAD (14, 26). This study overcomes this limitation; we found that compared with CPAP, MAD not only showed a consistent trend in its effect on nocturnal SBP but also demonstrated a significant advantage in reducing daytime SBP.

This study has several strengths. First, it included only randomized controlled trials, thereby minimizing the impact of potential confounding factors on the results. Second, most of the included studies used 24-h ambulatory blood pressure monitoring to assess blood pressure changes; this method is considered the gold standard for evaluating antihypertensive efficacy and overcomes the “white coat effect” commonly associated with clinic blood pressure measurements (40). Third, this study included multiple high-quality RCTs published in recent years, making the evidence more comprehensive and reliable.

However, this study still has certain limitations. First, there are significant differences in follow-up durations across studies; some studies had relatively short observation periods, which may underestimate the long-term effects of MAD on blood pressure. Second, the overall pooled blood pressure reductions are relatively modest, and there is substantial variability of response among individuals. This variability is strongly influenced by differences in OSA severity, baseline blood pressure levels, treatment adherence, and MAD device type across the included studies, all of which are factors that critically determine therapeutic effectiveness. Third, the included longitudinal studies exhibited significant methodological heterogeneity in evaluating hypertension outcomes. The considerable variations in blood pressure measurement techniques and adjustments for concurrent antihypertensive medications represent an important gap that may contribute to the variability in the observed effects. Fourth, objective adherence data were limited to only three recent trials, which warrants caution to avoid overinterpreting the role of adherence as the primary explanatory mechanism. Fifth, our literature search was restricted to publications in English, which may introduce a potential language bias. Finally, some studies had small sample sizes, limiting their statistical power. Future larger-scale, long-term follow-up randomized controlled trials are needed to further clarify the role of MAD in cardiovascular risk management for OSA patients.

Looking forward, OSA management is shifting from a “one-size-fits-all” paradigm toward precision medicine (41). Our findings underscore the pivotal role of the “efficacy-effectiveness balance,” wherein MAD's superior adherence compensates for its lower AHI reduction, yielding cardiovascular benefits comparable to CPAP. Building on this, emerging clinical trends strongly advocate for multimodality treatments. Combining MAD with positional therapy, hypoglossal nerve stimulation (42), or targeted pharmacotherapies (e.g., novel GLP-1/GIP receptor agonists like tirzepatide) (43) represents a highly promising frontier. Ultimately, these personalized, phenotype-driven strategies will address the multifactorial pathogenesis of OSA, optimizing adherence and maximizing long-term cardiovascular risk reduction.

## 5 Conclusion

This systematic review and meta-analysis indicates that MADs effectively improve sleep-related breathing events and clinical symptoms in patients with OSA, and show a trend toward better objective treatment adherence than CPAP. Regarding cardiovascular outcomes, MAD showed effects comparable to CPAP in overall blood pressure control and a statistically significant reduction in daytime SBP. Therefore, for OSA patients who cannot tolerate CPAP, MAD could be considered a viable alternative option for cardiovascular risk management.

## Data Availability

The original contributions presented in the study are included in the article/Supplementary material, further inquiries can be directed to the corresponding author.
