# Supplementary material for: Effects of mandibular advancement devices vs. CPAP on blood pressure in obstructive sleep apnea: a systematic review and meta-analysis of randomized controlled trials
Source: Front Neurol. 2026 May 20;17:1846726. doi: 10.3389/fneur.2026.1846726 (PMC13229622; doi:10.3389/fneur.2026.1846726)

Figure S1. Forest plot of 24-hour systolic blood pressure comparing MAD therapy versus inactive control.


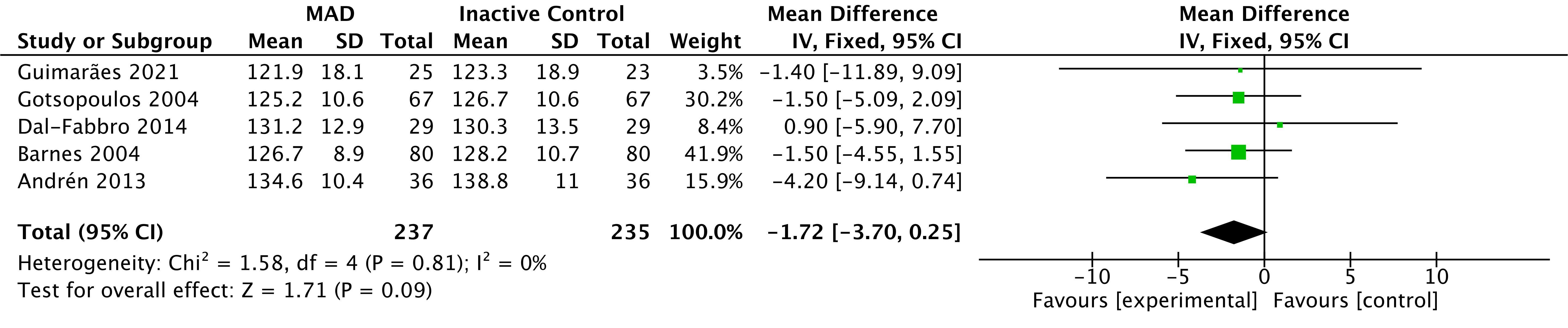


Figure S2. Forest plot of 24-hour diastolic blood pressure comparing MAD therapy versus inactive control.


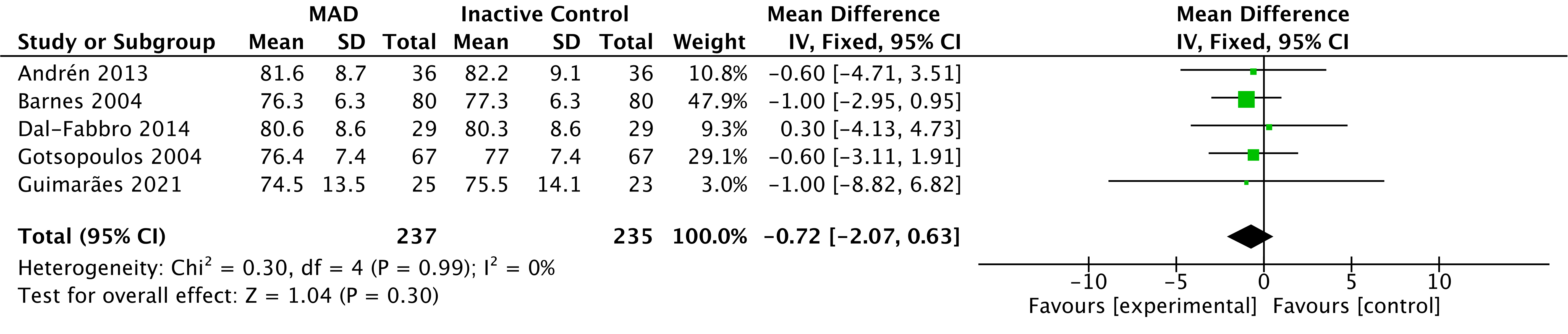


Figure S3. Forest plot of daytime systolic blood pressure (Daytime SBP) comparing mandibular advancement device (MAD) therapy versus inactive control.


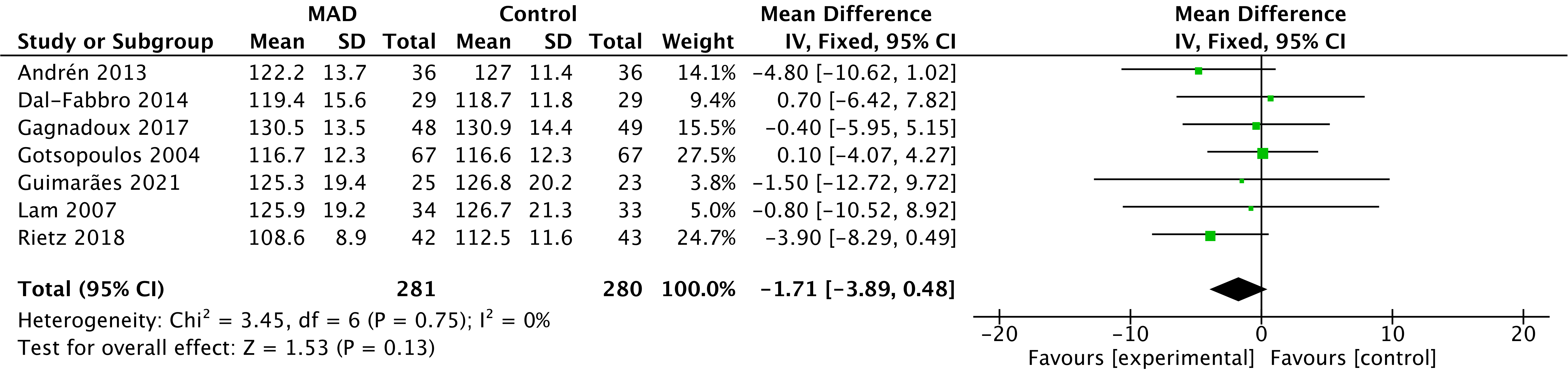


Figure S4. Forest plot of daytime diastolic blood pressure (Daytime DBP) comparing mandibular advancement device (MAD) therapy versus inactive control.


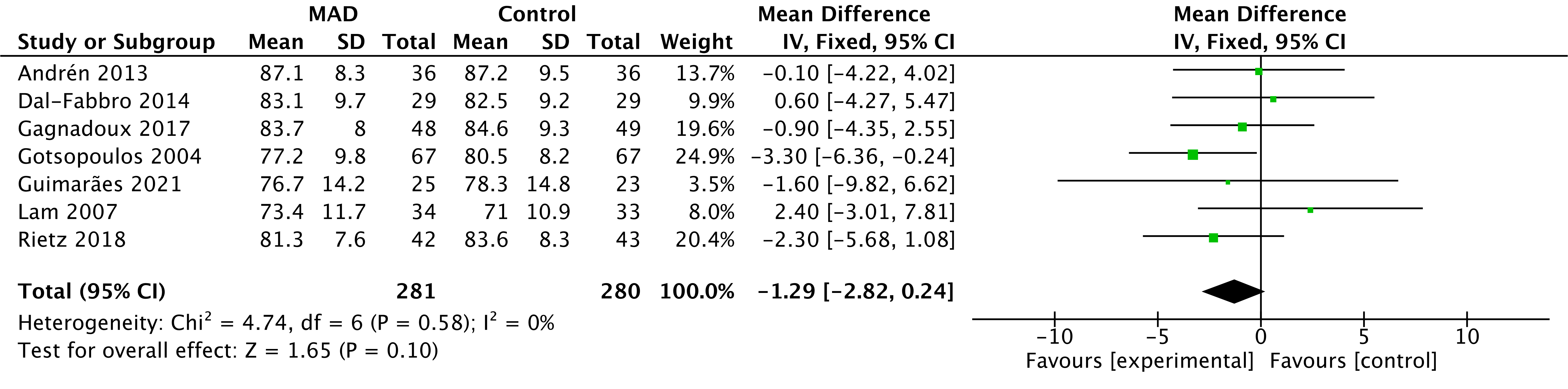


Figure S5. Forest plot of nighttime systolic blood pressure (Nighttime SBP) comparing mandibular advancement device (MAD) therapy versus inactive control.


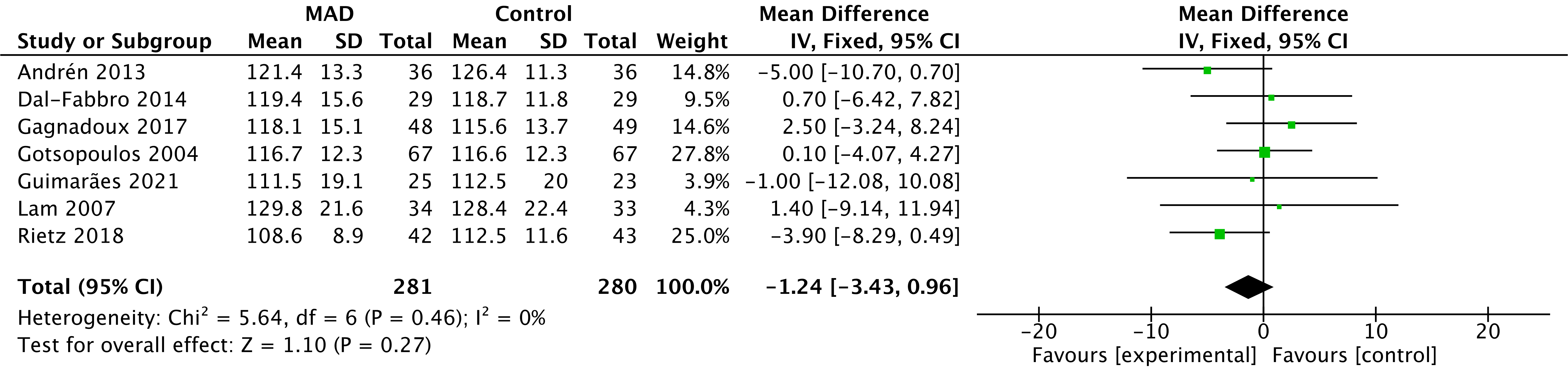


Figure S6. Forest plot of nighttime diastolic blood pressure (Nighttime DBP) comparing mandibular advancement device (MAD) therapy versus inactive control.


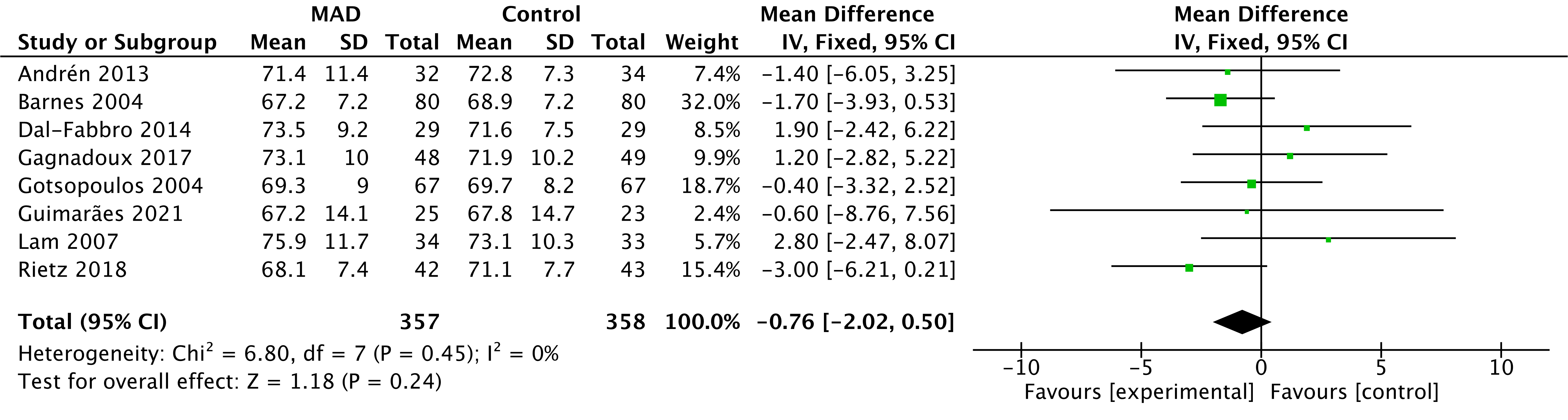


Figure S7. Forest plot of 24-hour systolic blood pressure (24-h SBP) comparing mandibular advancement device (MAD) therapy versus continuous positive airway pressure (CPAP).


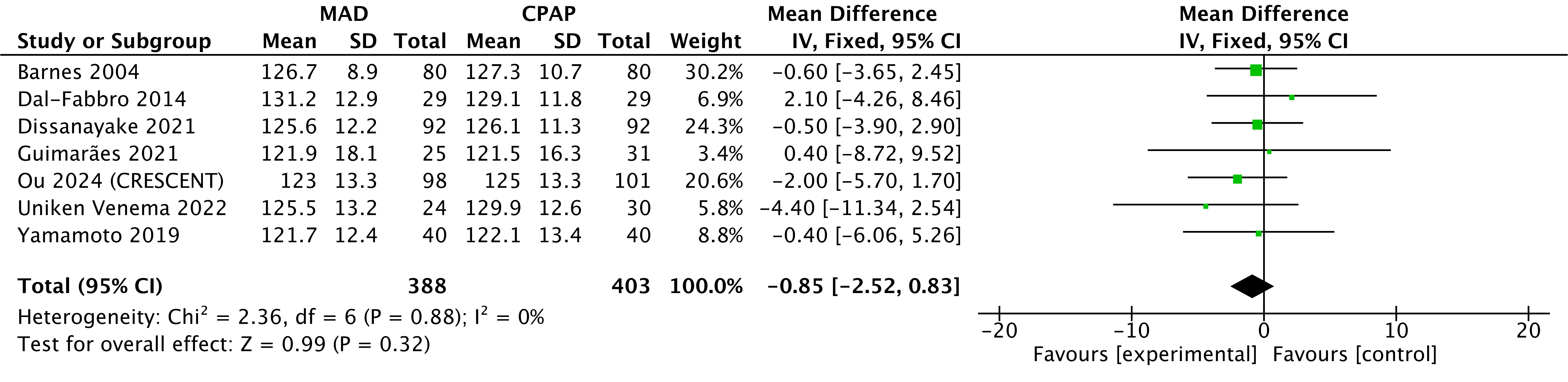


Figure S8. Forest plot of 24-hour diastolic blood pressure (24-h DBP) comparing mandibular advancement device (MAD) therapy versus continuous positive airway pressure (CPAP).


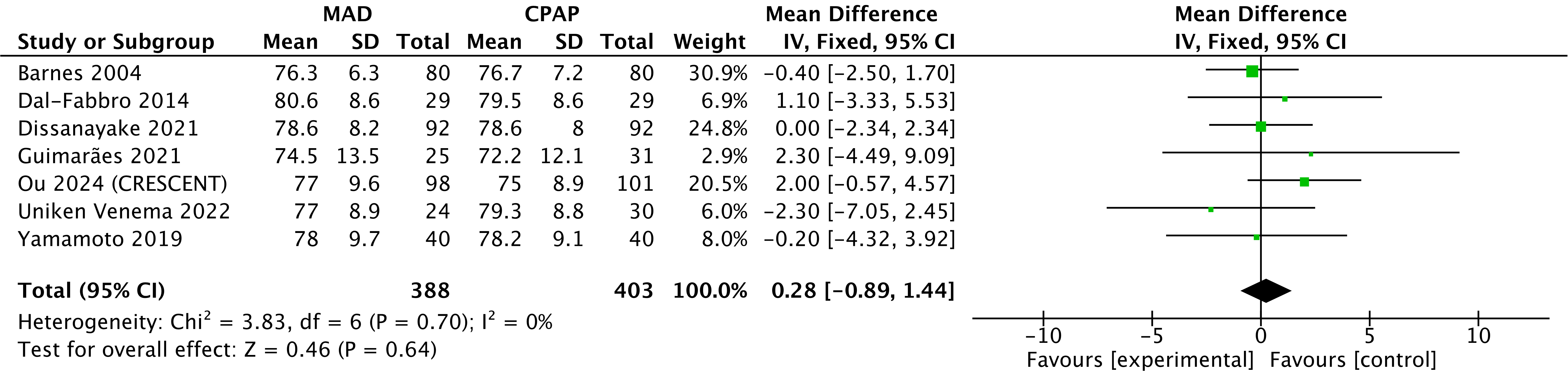


Figure S9. Forest plot of daytime diastolic blood pressure (Daytime DBP) comparing mandibular advancement device (MAD) therapy versus continuous positive airway pressure (CPAP).


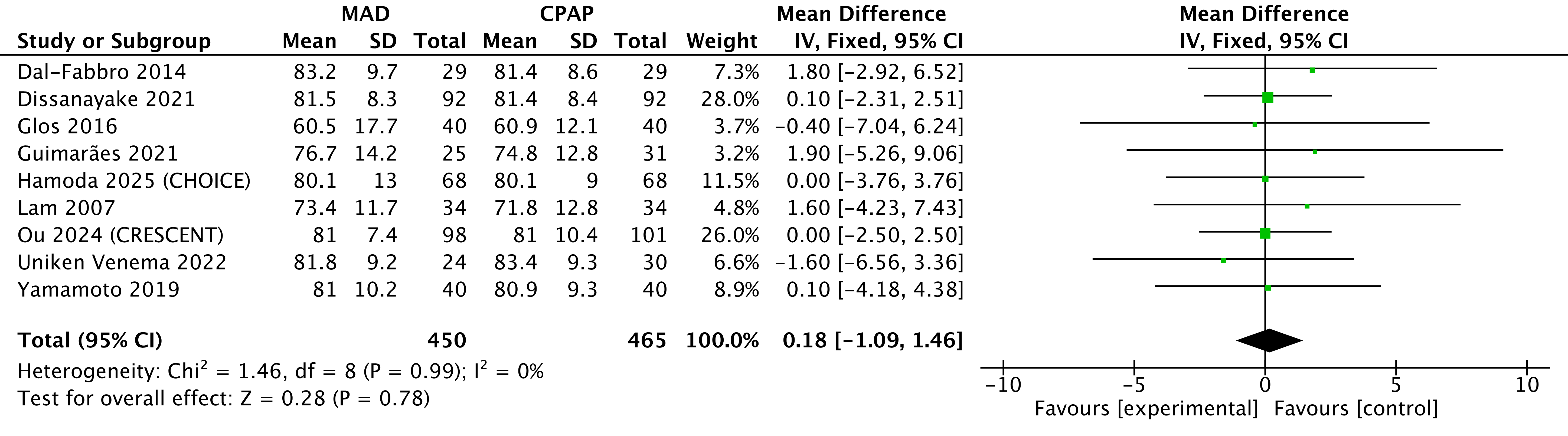


Figure S10. Forest plot of nighttime systolic blood pressure (Nighttime SBP) comparing mandibular advancement device (MAD) therapy versus continuous positive airway pressure (CPAP).


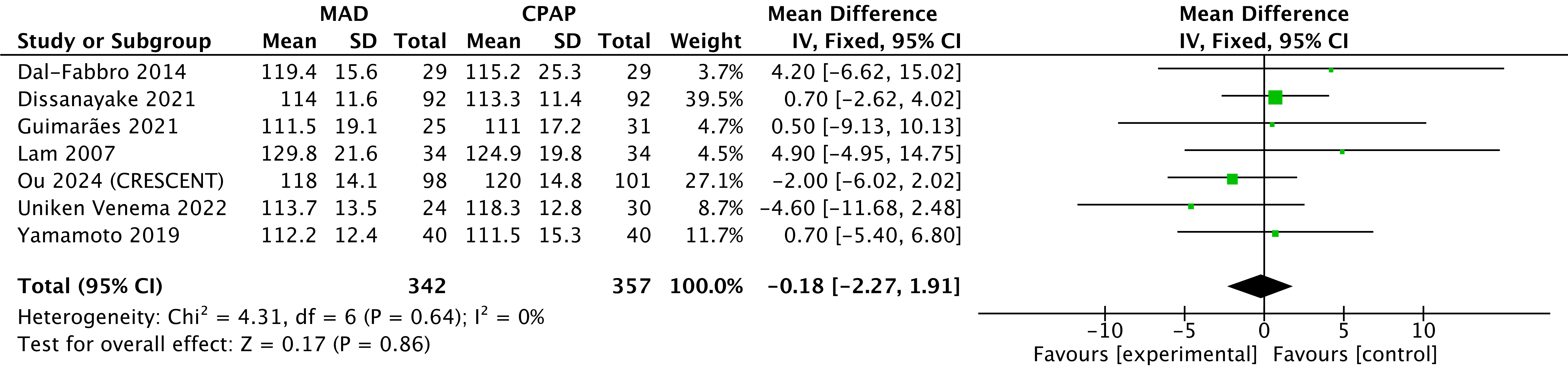


Figure S11. Forest plot of nighttime diastolic blood pressure (Nighttime DBP) comparing mandibular advancement device (MAD) therapy versus continuous positive airway pressure (CPAP).


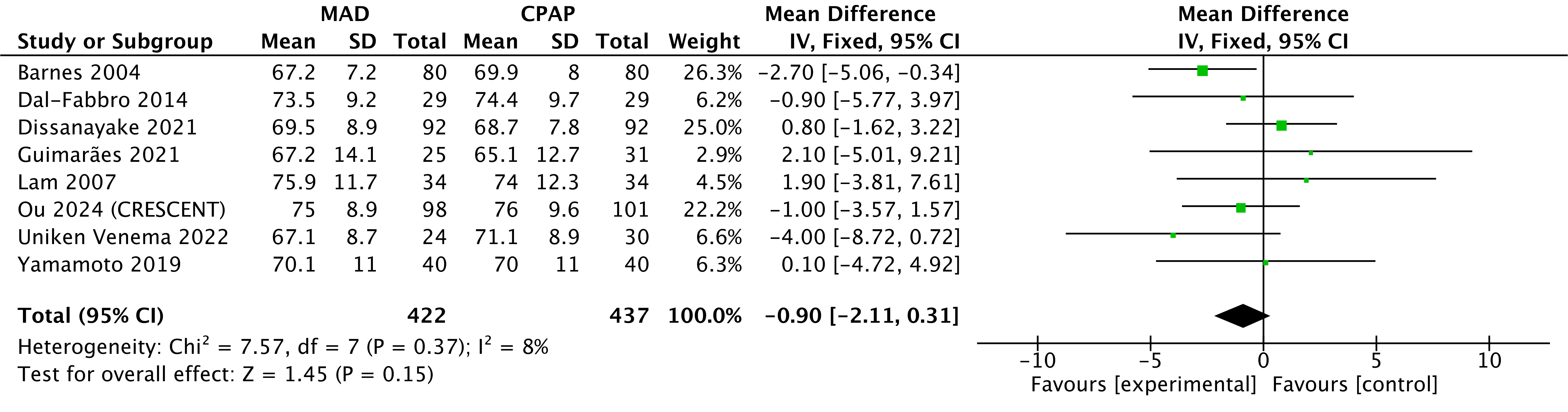


Figure S12. Forest plot of Epworth Sleepiness Scale (ESS) comparing MAD and CPAP.


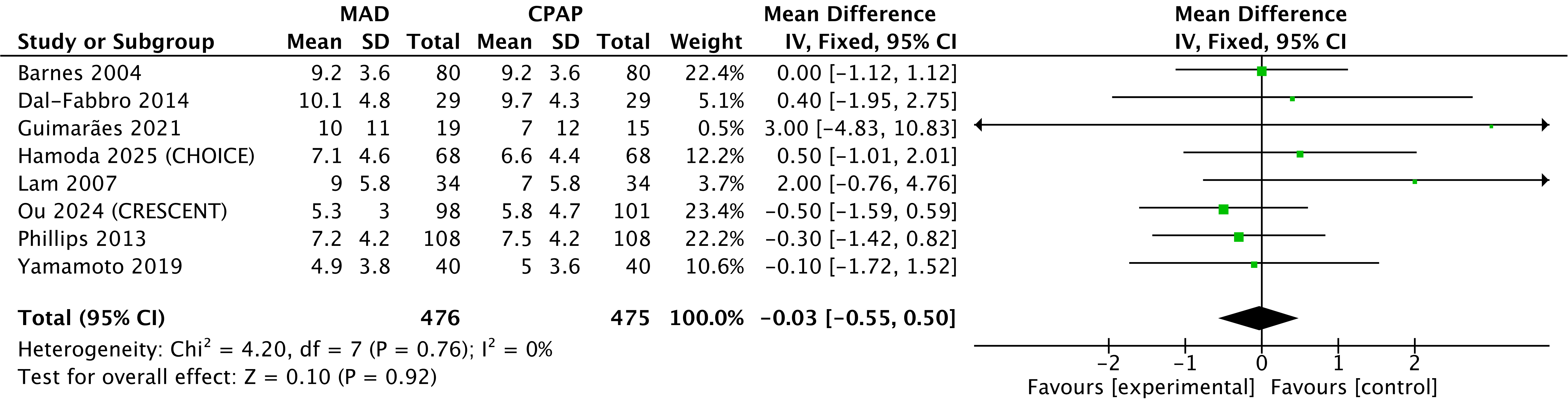

Supplement: Supplementary file 1 [file Table_1.docx]
